# Supplementary material for: Modelling the incremental benefit of introducing malaria screening strategies to antenatal care in Africa
Source: Nat Commun. 2020 Jul 30;11:3799. doi: 10.1038/s41467-020-17528-3 (PMC7393377; doi:10.1038/s41467-020-17528-3)
Supplement: Supplementary file 1 — Supplementary Information [file 41467_2020_17528_MOESM1_ESM.pdf]

Modelling the incremental benefit of introducing malaria screening strategies to antenatal care in Africa

Walker et al.

Supplementary Information

Supplementary Methods

The model used in this paper develops a previous model linking malaria transmission in the general population to exposure to malaria in pregnancy, accounting for prevalence and stage of placental infection, and also associated patterns of malaria-associated low birthweight (LBW) by gravidity. This model has previously been fitted to observed patterns of placental infection at delivery, by histological stage and gravidity, and the risk of LBW in the absence of intervention. Full details of the methodology used to fit the model to these data are described in full elsewhere[1,2]. Here we provide a succinct description of the model and fitted parameter distributions. We then describe how the model was used to infer the impact of pregnancy, and pregnancy-specific immunity, upon RDT sensitivity at first ANC visit during the second trimester, accounting for uncertainty in the unobserved level of exposure in previous pregnancies. Finally, we describe how parameters describing RDT sensitivity at subsequent ANC visits were also obtained and incorporated into the model and how these were the used to assess the effectiveness of different ANC-based prevention strategies.

Model linking patterns of exposure to malaria during pregnancy and malaria-attributable LBW to transmission within the general population.

Patterns of exposure to malaria in pregnancy (i.e. the prevalence of malaria at conception and subsequent infection rate throughout gestation) are linked to malaria transmission, as measured by the Entomological Inoculation Rate (EIR) – the number of infectious bites experienced by adults within the general population per year. This value is calculated using the equilibrium solution of a deterministic version of an established model of malaria transmission[3]. This model was specifically re-fitted for the purpose of better estimating the relationship between incidence and patterns of PCR prevalence outside of pregnancy by age and transmission[1], as these variables are likely to be most relevant for determining exposure to malaria in pregnancy.

Malaria transmission in the general population

In brief, non-pregnant individuals in the model are stratified by age category (a) and further sub-divided into 5 biting heterogeneity classes (h) to capture the variation in the number of mosquito bites received amongst the population. This results in the following heterogeneity- and age-strata specific force of infection, $\Lambda(h, a)$:

$$\Lambda(h, a) = E(h, a)b(h, a) \quad (1).$$

Here $E(h, a)$ describes the relationship between EIR, age and heterogeneity strata, given by:

$$E(h, a) = \mathrm{EIR}\left(1 - \rho \exp\left(\frac{a}{a_0}\right)\right)\varsigma(h) \quad (2)$$

Where $(1 - \rho)$ is the factor by which exposure to mosquitoes is lower in new-born babies (i.e. at age = 0) and a_0 determines the rate at which exposure increases with age. The size of the population in each of the $h = 1, .., 5$ heterogeneity strata and the relative biting rates of individuals in each biting heterogeneity class, $\varsigma(h)$, are equal to the weights and coefficients of the Hermite-Gauss

quadrature of a Log-Normal distribution with mean 1 and variance σ^2 to a best-fitting approximation of:

$$\log(\varsigma) \sim N\left(-\frac{\sigma^2}{2}, \sigma^2\right) \quad (3)$$

at the population-level. Meanwhile, $b(h,a)$ is the age-dependent probability of infection following an infectious bite which depends upon the level of "infection-blocking" immunity an individual has acquired.

Through this force of infection, Λ, the EIR then determines the proportion of the population at equilibrium in each age- and heterogeneity strata within 6 different infection states:

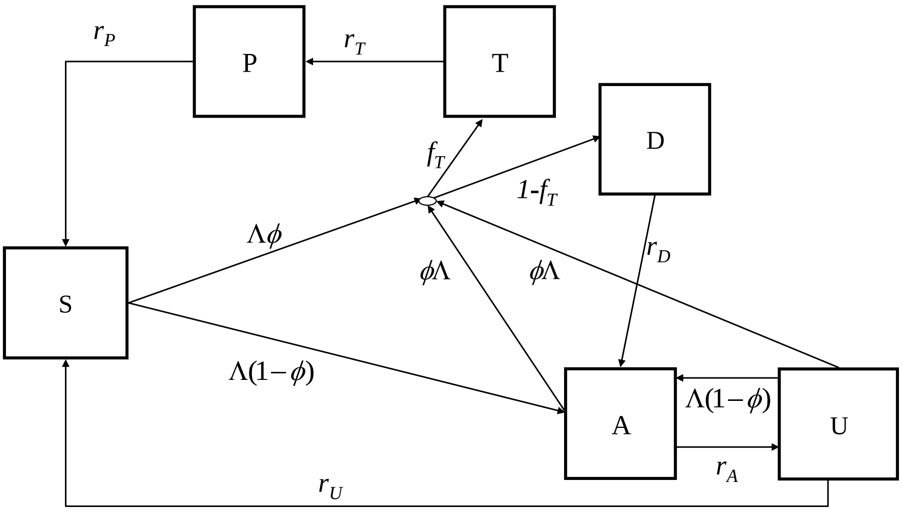

Supplementary Figure 1| Flow diagram for human stages of the general population model (taken from Griffin et al.[3].)

Individuals are born into a susceptible and parasite-free (S) stage within the first age-stratum of the hth heterogeneity class at a rate equivalent to the birth rate μ, multiplied by the hth Hermite-Gauss quadrature weighting. Individuals then become infected at a rate dependent on the age- and biting-heterogeneity specific force of infection $\Lambda(h,a)$. Upon infection, individuals experience clinical symptoms with probability $\phi(h,a)$, which depends upon the level of acquired immunity (see below), or they develop asymptomatic malaria (with probability $1-\phi(h,a)$) and enter state (A), where infection is detectable by slide microscopy. They then transfer to a lower density stage (U), in which infection is detectable only using PCR, according to a rate r_A, which depends upon acquired "blood-stage" immunity. They then clear infection and return to susceptible, parasite-free state (S) with rate r_U. Individuals who experience clinical symptoms are treated with probability f_t, entering a treatment stage (T), to account for post-treatment infectivity, before entering a non-infectious, protected stage (P), the duration of which depends upon the prophylactic profile of the treatment drug. Symptomatic individuals who are not treated enter an untreated disease stage (D), becoming asymptomatic at rate r_D, and entering stage (A). Individuals with asymptomatic infection (A, U) can become super-infected at rate $\Lambda(h,a)$ which can lead to symptomatic infection (T or D), or if they

are initially in stage (U), an increase in parasite density to levels detectable by microscopy, meaning a transition to stage (A).

Immunity in non-pregnant individuals is acquired in three ways:

1) Infection blocking immunity where the probability an infectious bite leads to a blood-stage infection within each age and heterogeneity category depends upon an immune level $I_B(h,a)$:

$$b(h,a) = b_{MIN} + \frac{b_{MAX} - b_{MIN}}{1 + (I_B(h,a)/I_{B0})^{\kappa_B}} \quad (4).$$

Here b_{MAX} is the probability of infection following an infectious bite within an immunologically naïve person, b_{MIN} is the probability of infection following an infectious bite in a person with maximum immunity. I_{B0} and κ_b are scale and shape parameters of the Hill function determining how the acquisition of immunity $I_B(h,a)$ moves individuals between these extrema with age.

$I_B(h,a)$ is assumed to increase in proportion to the entomological inoculation rate within a age and biting heterogeneity strata, $E(h,a)$, raised to the fitted power parameter τ. It is assumed to wane at a fixed rate $1/w_b$:

$$\frac{dI_B}{da} = E(h,a)^{\tau} - \frac{I_B}{w_B}, \quad I_B(h,0) = 0 \quad (5)$$

2) Blood-stage immunity which serves to reduce the duration of time in which new infection remains above microscopically-detectable density by increasing the rate at which individuals progress from A to U:

$$r_A(h,a) = r_{A0}\left(1 + (r_{AMAX}/r_{A0} - 1)\frac{(I_A(h,a)/I_{A0})^{\kappa_A}}{1 + (I_A(h,a)/I_{A0})^{\kappa_A}}\right). \quad (6)$$

Here the rate of progression in non-immune individuals $r_{A0} = 1/(d_I - d_D)$, where d_I is the duration of time untreated non-immune individuals retain densities of parasitaemia detectable by microscopy (including with symptoms) and d_D is the duration of time in which clinical symptoms persist. $r_{AMAX} = \frac{1}{d_{AMIN}}$, represent the rate of progression to sub-microscopic infection in individuals with maximum immunity, parameterised according to its reciprocal, the average duration of slide positivity in these individuals. I_{A0} and κ_A are scale and shape parameters of the Hill function determining how the acquisition of immunity $I_A(h,a)$ moves individuals between these extrema with age, with I_A assumed to decay with age according to a constant rate w_A:

$$\frac{dI_A(h,a)}{da} = 1 - \frac{I_A(h,a)}{w_A}, \quad I_A(h,a) = 0 \quad (7)$$

3) Clinical immunity. The development of immunity to modulate the probability of acquiring clinical disease upon infection by age is also modelled according to a hill function with shape and scale parameters I_{C0} and κ_C:

$$\phi(h,a) = \frac{1}{1 + (I_C(h,a)/I_{C0})^{\kappa_C}} \quad (8)$$

Where $I_C(h,a)$ is driven by a combination of acquired and maternally derived immunity, immunity that protects children in early life, represented by $I_{CA}(h,a)$ and $I_{CM}(h,a)$, where $I_{CA}(h,a)$ is driven by the force of infection and a decay parameter w_{CA}:

$$\frac{dI_{CA}(h,a)}{da} = \frac{\Lambda(h,a)}{1+\Lambda(h,a)} - \frac{I_{CA}(h,a)}{w_{CA}}, \quad I_{CA}(h,a) = 0, \quad (9)$$

whereas I_{CM} at birth is assumed to represent a proportion P_{CM} of immunity in young adults and a waning parameter w_{CM}:

$$\frac{dI_{CM}(h,a)}{da} = -\frac{I_{CM}(h,a)}{w_{CM}}, \quad I_{CM}(h,a) = P_{CM} I_{CA}(h,20) \quad (10)$$

Supplementary Table 1 Fitted parameters and prior and posterior distributions for the parameters from the general population model

Parameter	Description (units)	Posterior Median (95% credible interval)*	Source[¥]
**Age and heterogeneity**			
ρ	Proportion of biting exposure which is age-dependent	0.85 (F)	[4,5] [[3]]
a_0	Rate parameter for increase in exposure with age	2920 days (F)	
σ^2	Variance of log of heterogeneity in biting rates	1.04 (0.83-1.27)	[1]
**Infectious periods**			
d_I	Duration of slide-positivity in non-immune individuals	200 days (F)	[6,7][[3]]
d_D	Duration of symptomatic infection	5 days (F)	[7][[3]]
$d_U = 1/r_U$	Duration of sub-microscopic infection	107.9 (92.3-122.8) days	[1]
$d_T = 1/r_T$	Duration of post-treatment infectivity	5 days (F)	[8][[3]]
$d_P = 1/r_P$	Duration of post-treatment prophylaxis	9 days (F)[†]	[9]
**Immunity parameters**			
b_{MAX}	Probability of human infection from an infectious bite with no immunity	0.93 (0.84-0,98)	[1]
b_{MIN}	Probability of human infection from an infectious bite with full immunity	0.006 (0.004,0.01)	[1]
I_{B0}	Infection blocking immunity scale parameter	1072.1 (916.3, 1231.5)	[1]
κ_B	Infection blocking immunity shape parameter	6.05 (5.17, 7.21)	[1]
τ	Rate of acquisition of Infection blocking immunity power scaling parameter	0.21 (0.17-0.24)	[1]
w_B	Decay parameter for infection blocking immunity	10 years	[[3]]
$d_{AMIN} = 1/r_{AMAX}$	Duration of slide-positivity in fully-immune individuals	160 days (F)	[10][[3]]
I_{A0}	Blood-stage immunity scale parameter	4732.5 (F)	
κ_A	Blood-stage immunity shape parameter	5 (F)	
w_A	Decay parameter for blood-stage immunity	10 years (F)	
I_{C0}	Protection from clinical disease scale parameter	53.03 (41.65,66.64)	[1]
κ_c	Protection from clinical disease shape parameter	2.01 (1.54,2.80)	[1]
w_{CA}	Decay parameter for acquired clinical immunity	30 years	[[3]]
P_{CM}	Immunity level of new-born relative to mother	0.53 (0.25,0.88)	[1]
w_{CM}	Decay parameter for maternal immunity	230.4 (143.9,348.0)	[1]

*Parameters which were fixed, either on the basis of original sources or due to issues of identifiability within model fitting are marker with an (F)
¥Sources of posterior distributions refer to the manuscript in which the parameter was fitted, for fixed parameters both the primary source for the value, if applicable, and the first manuscript in which this was used (in brackets) are given.
†On the basis of Artemether-lumefantrine which provides protection for a duration ($d_T + d_P$) of around 14 days[9].

Exposure to peripheral and placental malaria infection and the risk of low-birthweight during pregnancy

The model of exposure to malaria in pregnancy is individual-based. Each simulated woman is assigned a heterogeneity class H at age 15. Age at each conception throughout her lifetime is denoted $C = \{C_g\}$, where $g = 1, .., F$ and F represents the total number of pregnancies by age 50. These values are generated according to gravidity-specific fertility rates stratified by 5 year age strata between 15-49 calculated from DHS surveys (https://dhsprogram.com/).

Peripheral infections

The times at which a woman is exposed to peripheral infection during her gth pregnancy $B_g = \{B_{g,i}, i = 1..n_B\}$, where n_B is the total number of blood-stage infections, and the times at which these infections clear $K_g = \{K_{g,i}, i = 1..n_B\}$, are then tracked throughout gestation. The infection status at the beginning of pregnancy, I_0 drawn according to the proportion of the population in each infection stage for that age- and biting-heterogeneity class such that $B_{g,1} = 0$, (i.e. the time of infection is the same as day 0, the day of conception) with probability $\frac{D(H,C_g)+A(H,C_g)+U(H,C_g)}{N(H,C_g)}$, the prevalence within the strata as detected by PCR for asexual-stage parasitaemia.

If not infected at conception, $B_{g,1} = X(\Lambda(H, C_g))$, where $X(.) \sim Exp(.)$. Subsequent blood stage infections are then drawn according to the force of infection experienced throughout pregnancy:

$$B_{g,j+1} = B_{g,j} + X(\Lambda(H, C_g)) \quad (11)$$

B_g is then the set of $B_{g,j} < 280$ (i.e. 40 weeks, the assumed timing of delivery). If $B_{g,1} = 0$, (i.e. the woman was infected at the point of conception), the time during gestation at which this infection will eventually clear, if at all, depends upon the stage the infection had reached:

$$K_{g,1} = \begin{cases} X(r_D) + X\left(r_A(H, C_g)\right) + X(r_U) & \text{if } \mathrm{I}_0 = D \\ X\left(r_A(H, C_g)\right) + X(r_U) & \text{if } \mathrm{I}_0 = A \\ X(r_U) & \text{if } \mathrm{I}_0 = U \end{cases} \quad (12)$$

For subsequent infections ($j > 1$) clearance times depend on the time of exposure and the level of acquired immunity:

$$K_{g,j} = \begin{cases} B_{g,j} + X(r_D) + X\left(r_A(H, C_g)\right) + X(r_U) & \text{with probability } \phi(H, C_g) \\ B_{g,j} + X\left(r_A(H, C_g)\right) + X(r_U) & \text{otherwise} \end{cases} \quad (13)$$

(N.B. though not included in the above equations for ease of notation, symptomatic infections are also treated with probability f_t, in which case they contribute to neither B_g nor K_g and a period of prophylaxis equivalent to $X(r_T) + X(r_P)$ is incorporated into the calculation of these times).

Placental infections

Placental malaria consists of malaria parasites becoming 'sequestered' in the placenta. This stage is responsible for much of the morbidity specific to malaria in pregnancy. We assume that this can occur at any point during a peripheral infection from 12 weeks gestation (around the time maternal blood begins to flow readily into the intervillous space of the placenta[11]). We assume that it takes a week (i.e. 2-3 cycles of replication) for parasites to begin expressing VAR2CSA, the Plasmodium falciparum erythrocyte membrane protein 1 (PfEMP-1) variant which binds to Chondroitin Sulphate A (CSA) and reaching appreciable densities within the placenta (i.e. the earliest placental infection could occur is 12+ 1 = 13 weeks after conception).

In primigravidae, or pregnant women who have not been exposed to placental infection previously we assume that all infections are capable of sequestering to densities within the placenta that would be detectable by histological analysis (note such analysis is only generally possible at delivery). This assumption is based upon previous modelling where evidence of placental infection (including pigment left by resolved infection) at delivery were estimated to be consistent with expected cumulative incidence of exposure given contemporary estimates of local EIR[1].

If a woman has a peripheral infection prior to 12 weeks of gestation placental infection will therefore occur at 13 weeks of gestation, provided the peripheral infection lasts beyond 13 weeks of gestation. Define $P_g = \{P_{g,i}, i = 1 \dots n_P\}$ as the timings at which n_P separate infections sequester within the placenta. In primigravidae, in the absence of intervention, $P_{1,1} = 91$ (i.e. 13 weeks gestation) if

$$\sum_j \left[1\left(\mathrm{B}_{1,j} < 84 \,\&\mathrm{K}_{1,j} > 91\right)\right] \geq 1 \quad (14)$$

where $1(.)$ represents the indicator function, taking the value 1 if true and 0 if false. After this initial stage of susceptibility of infection, subsequent infections sequester within the placenta a week after any new blood-stage infection (i.e. $\mathrm{B}_{1,j} + 7$).

Once sequestered, a placental infection in the model can go through three stages of infection, each corresponding to distinct stages of infection that can observed at delivery through histology. Histological examination looks at two aspects, the presence of parasites (referred to as 'active' infection), and the presence of detectable hemozoin pigmentation (a by-product of parasite digestion of red blood cells).

1) acute infection, where parasites are present in the placenta, but have not been sequestered for sufficient time or at sufficient density to produce detectable hemozoin pigment;
2) chronic infection, where parasites are present AND pigmentation is detectable either freely or trapped within fibrinoid deposits;
3) past infection, where no visible parasites remain but pigment remains visible (note as we only consider active infection in this analysis this aspect of the model is not described).

The set of times at which each placental infection is cleared from the placenta, $R_g = \{R_{g,i}, i = 1 \dots n_P\}$, are then:

$$R_{g,i} = P_{g,i} + X(\gamma_1) + X\left(\gamma_C\left(\nu_g\right)\right) \quad (15)$$

where γ_1 is the rate at which placental infections that would be diagnosed as acute by histology at delivery produce sufficient pigment to be diagnosed as chronic, and $\gamma_C\left(\nu_{i,g}\right)$ is the rate at which these chronic infections are cleared so that only pigment would be detected, which depends upon

the number of times a women has been infected in pregnancies preceding the current one ν_g as described below.

Immunity to placental malaria

Immunity to placental parasites is acquired over successive pregnancies. We assume this is acquired as a function of the number of previous exposures to placental infection a woman has experienced in previous pregnancies $\nu_{i,g}$. This can affect the progression of placental sequestration in two ways (representing the best fitting of a range of models fitted to patterns of placental histology in the absence of intervention[1]):

1) it can lead to a peripheral infection failing to sequester within the placenta to appreciable levels (and thus is not included within $\mathrm{P}_{i,g}$) with probability:

$$\frac{1}{\left(1+\left(\frac{\nu_g}{\xi}\right)^{\alpha}\right)} \quad (16)$$

   where ξ and α are shape and scale parameters of this "placental infection blocking immunity" which were fitted to the histology data.

2) It also serves to increase the rate of clearance of chronic infection:

$$\gamma_C(\nu_g) = \gamma_{C0}\left(1+\left(\frac{\nu_g}{\pi}\right)^{\psi}\right) \quad (17)$$

   where π and ψ are shape and scale parameters of this "chronic placental infection-stage immunity" and γ_{C0} is the rate of clearance in primigravid women, or multigravidae who were never exposed during previous pregnancies.

The posterior distribution of these parameters were then fitted to data on the prevalence of placental infection at each stage in different settings and by gravidity in the absence of intervention and are presented in Supplementary Table 2 (see[1] for full details of model fitting).

PCR positivity throughout pregnancy, x_g^P, is then defined according to whether any infection remains active, either within the peripheral blood or placenta:

$$x_g^P(t) = 1\left(\left[\sum_{j=1}^{n_B}\left[1\left(\mathrm{B}_{g,j} < t, \mathrm{K}_{g,j} > t\right)\right] + \sum_{j=1}^{n_P}\left[1\left(P_{g,i} < t, R_{g,i} > t\right)\right]\right] \geq 1\right) \quad (18)$$

Supplementary Table 2| Fitted parameters for the model of placental infection

Symbol	Description	Posterior Median (with 95% credible interval)	Source
$1/\tau_A$	Mean duration of acute infection (days)	53.7 (41.2,65.2)	1
$1/\tau_C$	Mean duration of chronic infection in previously unexposed women (days)	94.2 (73.5,118.1)	1
ν	Power parameter of infection blocking immunity	1.01 (0.51,2.36)	1

ξ	Offset power of infection blocking immunity	14.5. (8.3,37.4)	1
ψ	Power parameter of faster clearance of chronic infection	2.23 (0.59,9.68)	1
π	Offset parameter of faster clearance of chronic infection	1.16 (0.31,1.98)	1
ζ	Additional hazard of LBW per day with chronic infection	0.002 (0.001, 0.003)	2

Exposure to infection and Low Birth Weight (LBW)

In a subsequent analysis[2], various models of the link between exposure to infection and malaria attributable LBW were fitted to data from studies describing the pattern of LBW, stratified by gravidity and stage of placental infection at delivery. The best fitting model, among those fitted, was one in which the risk of LBW is driven by the duration during which a women experiences exposure to the chronic stage of placental infection. The additional risk of LBW is accumulated according to a constant per-capita additional risk of LBW multiplied by the duration of gestation in which chronic infection occurred.

Inferential framework for estimating the impact of pregnancy upon RDT sensitivity

Inference upon the set of parameters determining the impact of pregnancy upon RDT sensitivity, denoted Ψ, was conduced within a Bayesian framework. We fitted the model linking transmission in the general population and exposure to malaria in pregnancy, with parameters Θ listed in Supplementary Tables 1 and 2, to the number of women testing positive by PCR in pregnancy by gravidity at enrolment within each trials. Specifically, fitting the probability of being PCR positive during any pregnancy $\mathrm{PCR}^P = \{\mathrm{pcr}^P_{g,j}\}$, to obtain the unobserved PCR prevalence within non-pregnant adults in each trial, $\mathrm{PCR}^A = \{\mathrm{pcr}^A_j\}$, and the probability distribution, $V_{g,j}(y)$, of the unobserved number of previous pregnancies y each women has experienced previously, which depends upon the level of transmission in site j and the woman's gravidity g. This was used to fit Ψ to the set of RDT results in each setting stratified by gravidity, $\mathrm{RDT}^P = \{\mathrm{rdt}^P_{g,j}\}$, as follows:

$$P(\Psi, E|\mathrm{RDT}^P, \mathrm{PCR}^P, \Phi, \Theta, \mathrm{F}) \propto P(\mathrm{RDT}^P|\Psi, \mathrm{PCR}^P, \Phi, E, \Theta, \mathrm{F})P(\mathrm{PCR}^P|E, \Theta, \mathrm{F})P(\mathrm{E}, \Psi) \quad (19)$$

PCR prevalence in pregnant women of gravidity g within site j was modelled based upon the simulated lifetimes of 1 million women. Here, pregnancies are assuming to occur at age- and gravidity-specific rates which are calculated from the most recent national DHS survey for the first administrative unit in which the trials took place (these rates are denoted $F = \{F_{a,g,j}\}$). Defining this PCR prevalence $\bar{x}_{g,j}$:

$$P(\mathrm{PCR}|E, \Theta, \mathrm{F}) = \prod_j \prod_g \bar{x}_{g,j}{}^{pcr_{g,j}} \left(1 - \bar{x}_{g,j}\right)^{N_{g,j} - \mathrm{pcr}_{g,j}} \quad (20)$$

a binomially-distributed likelihood linking observed PCR prevalence to that generated by the model, where $N^P_{g,j}$ represents the number of women tested at enrolment stratified by site and gravidity. The likelihood of observing the number of RDT positive test results within those women who were PCR positive is then

$$P(\mathrm{RDT}|\mathrm{PCR}, \Psi, \Phi, E, \Theta, \mathrm{F}) = \prod_j \prod_g \bar{s}_{g,j}(\Phi)^{\mathrm{rdt}_{g,j}} \left(1 - \bar{s}_{g,j}(\Phi)\right)^{\mathrm{pcr}_{g,j} - \mathrm{rdt}_{g,j}} \quad (21)$$

where $\bar{s}_{g,j}(\Phi)$ is the average RDT sensitivity within women of gravidity g in site j.

We examined four possible functions for $\bar{s}_{g,j}(\Phi)$ representing: **Model 1:** that sensitivity is independent of transmission and immunity; **Model 2:** that the odds of detecting infection are proportional to those in non-pregnant adults, which in turn depends upon transmission, but are independent of gravidity, **Model 3:** that the odds of detecting infection decline with gravidity-dependent immunity but are independent of transmission setting; **Model** 4**:** that the odds in primigravidae are proportional to those in non-pregnant adults but then decline with gravidity - specific immunity – parameterisations of these models are shown in Supplementary Table 3.

Supplementary Table 3| Model comparison of different models of the impact of pregnancy upon RDT sensitivity at enrolment into ISTp.

Model	Parameterisation	Deviance	pD	DIC
**1. Transmission and gravidity independent**	$\text{Odds}(S_{ij}^{P}) = \beta$	5554.78	5.4	5559.64
**2. Transmission dependent, gravidity independent**	$\text{Odds}(S_{ij}^{P}) = (1+\beta)\,\text{Odds}\left(S^{A}(x_j)\right)$	5500.22	6.1	5506.32
**3. Transmission dependent, gravidity dependent**	$\text{Odds}(S_{ij}^{P}) = \left(\frac{\beta}{(1+y_{ij}/\delta)^{\mu}}\right)$	5414	7.68	5421.68
**4. Transmission-, gravidity-dependent**	$\text{Odds}(S_{ij}^{P}) = \left(1+\frac{\beta}{(1+y_{ij}/\delta)^{\mu}}\right)\text{Odds}\left(S^{A}(x_j)\right)$	5365.12	8.2	5370.24

Models were fitted using Markov Chain Monte Carlo (MCMC), with parameters updated in isolation using a Metropolis-Hastings proposal distribution with standard deviation tuned to a targeted 23% acceptance ratio during the burn-in of 10,000 iterations, chains were then run for a further 100,000 iterations, with auto-correlation reduced by selected draws from the posterior every 10th iteration. Convergence was assessed visually and by starting chains from multiple initial values. Model selection was based upon the Deviance Information Criterion (see Supplementary Table 3), on this basis Model 4 was selected. Posterior distribution for the parameters determining the impact of pregnancy upon RDT sensitivity, as well as the level of transmission within each trial setting are shown in Supplementary Figure 1 and Supplementary Table 4. The fit of the model to the data is shown in Figure 2 of the main text.

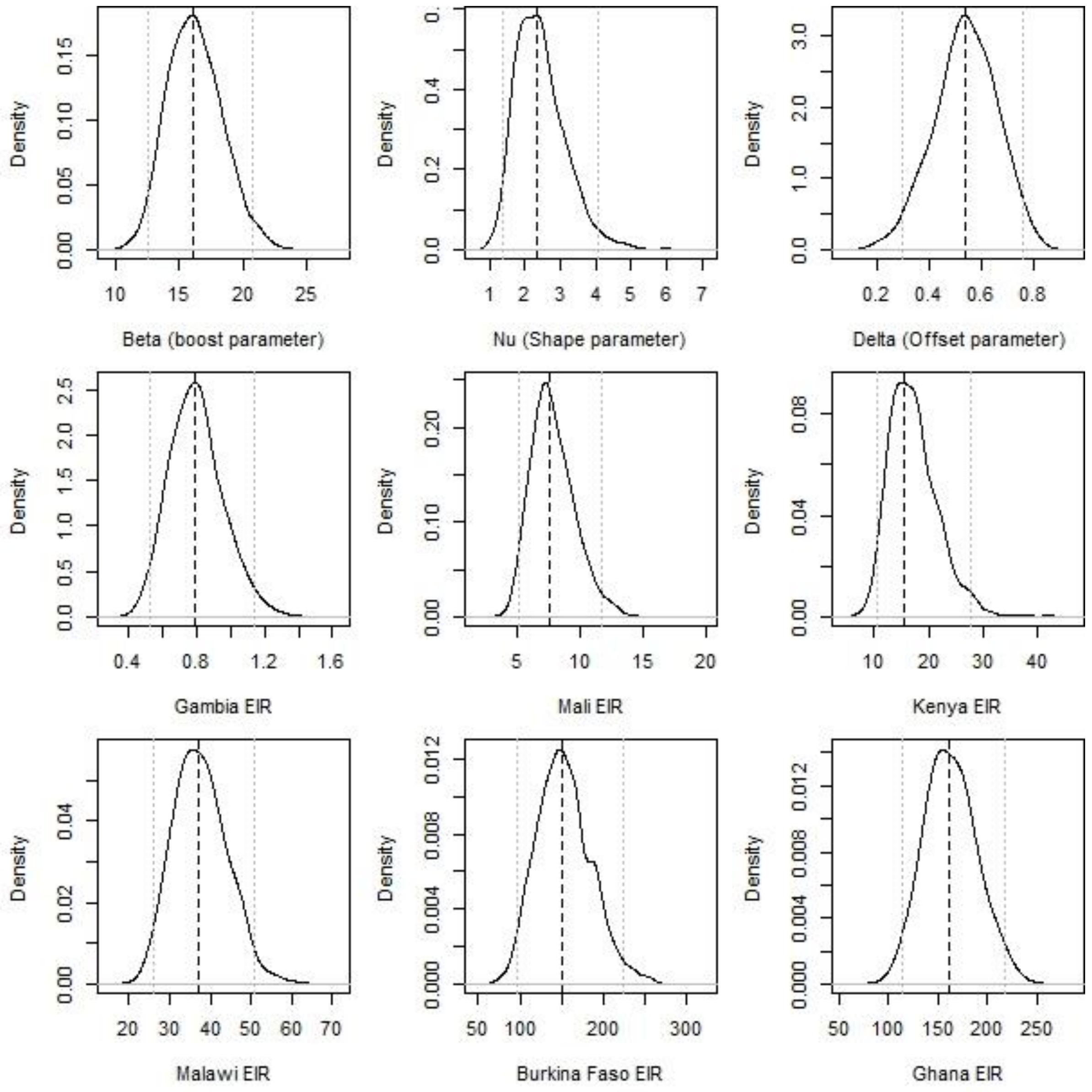

Supplementary Figure 2| Posterior distribution for the final fitted model of RDT sensitivity (Model 4). Top row shows parameters describing the impact of pregnancy upon immunity, bottom two rows show site-specific EIR. Black dashed lines show posterior medians, grey dotted lines show 95% credible intervals.

Supplementary Table 4| Prior and posterior distributions for the final fitted model of RDT sensitivity (Model 4).

Parameter	Prior distribution (parameters)	Posterior distribution Median [95% credible interval]
$\beta -$ **boost in odds of detection relative to non-pregnant adults**	Gamma (shape =0.001, scale=1000)	16.06 [12.50-20.77]
$\mu -$ **shape parameter for effect of gravidity-specific immunity**	Gamma (2,5)	2.35 [1.39-4.06]
$\delta -$ **offset parameter for effect of gravidity-specific immunity**	Gamma (2,0.4)	0.54 [0.30-0.76]

**Trial specific annual EIR:**	Uniform (0,10000)	
**Burkina Faso**		150.8 (97.3-223.6)
**The Gambia**		0.79 (0.53-1.14)
**Ghana**		161.8 (114.6-216.6)
**Kenya**		16.7 (10.5-27.7)
**Mali**		7.61 (5.16-11.73)
**Malawi**		37.1 (26.2-50.67)

Incorporating the sensitivity of RDTs during subsequent visits

Infections at subsequent visits following enrolment reflect some combination of (i) new infections, (ii) infections that were not detected at the previous visit and have persisted throughout the interim period and (iii) infections that were detected but were not successfully cleared. As a result, RDT sensitivity at later visits is unlikely to be related to sensitivity outside of pregnancy in any straightforward way. We therefore modelled RDT sensitivity at subsequent visits separately using logistic regression. Potential individual-level variables considered within the regression were: gravidity; number of previous visits where testing had been conducted; PCR result at previous visit; RDT result at previous visit; whether infection had previously been detected throughout pregnancy by RDT; whether infection had previously been detected throughout pregnancy by PCR. Model selection was based upon stepwise and reverse-stepwise regression, including a normally-distributed random-effect term for each site. The best fitting model by AIC included an intercept term representing an odds of detecting infection in primigravidae not infected at the previous visit of 1.14 (0.52-2.52 95% Confidence Interval (C.I.)) this is then modified by gravidity, with a decrease in odds ratio (OR) = 0.87 (0.78-0.96 95% C.I., p=0.005) per pregnancy, and if the woman was positive by PCR at her previous visit, OR= 0.70 (0.53-0.92, 95% C.I., p=0.01). Significant heteroskedasticity between study sites remained unaccounted for within this final model, which may reflect variation in spacing or timing of ANC visits, biological and immunological interactions or drug adherence that we were not able to capture. However, comparison of model simulations of dynamics of PCR prevalence throughout pregnancy and those observed in the data suggest a good overall agreement (see Figure 3 of the main text).

Implementing alternative strategies within the model

We define the set of ANC visits $V = \{v \in 1..n_v\}$, with associated timings for each ANC visit t_v. The modelled sensitivity of RDT if provided at each visit, which depends upon the gravidity, visit number and previous infection status of a women at the visit as described above, is then denoted s_v. The result of an RDT at visit v was then positive if the woman was PCR positive at the time of the visit (i.e. $x_g^P(t_v) = 1$) and $s_v > u_{v,1}$, where $u_{v,1}$ is a random draw from a uniform distribution between 0 and 1. Women then either received no intervention, the same drug regardless of the result (IPTp), a drug only if they tested positive (ISTp) or a different drug dependent upon whether they were positive or negative (hybrid). Each drug was assigned a curative efficacy parameter e_D, representing the probability the drug was effective at clearing an infection, and an average duration of prophylaxis hl_D as described in the main text (in the cases of SP this depended upon the level of SP resistance within the simulation). If a drug was provided it was effective if $e_D > u_{v,2}$ (a second draw from a uniform distribution between 0 and 1).

If effective treatment was implemented by truncating any ongoing infections:

$$K_{g,j} = t_v \qquad \forall j \; s.t. (K_{g,j} > t_v \cap \; B_{g,j} < t_v) \quad (22)$$

A period of prophylaxis $\rho_v \sim W(hl_D, 8)$, where $W(a, b)$ is a Weibull distribution with mean a and shape parameter b. (NB, This distribution was chosen to replicate a more realistic distribution of prophylactic duration whereby a drug provides good protection until a threshold minimum inhibitory drug concentration is reached, and afterwards protection declines rapidly, relative to a more commonly used exponential distribution which, due to it high variation, is likely to overestimate the proportion of women who receive full protection between visits). This period of protection was then implemented by pruning any new infections occurring in the interval $[t_v, t_v + \rho_v]$, as well as any placental exposure associated with that infection.

Uncertainty analysis of the relative impact of alternative strategies

An uncertainty analysis of the relative effectiveness of the alternative strategies to IPTp-SP in an area with high quintuple resistance was conducted (see Figures 5 and 6 of main text) which included the main factors determining incremental effectiveness within the model. This involved simulating from 300 draws of the fitted joint posterior of our model of the natural progression of malaria during pregnancy (Supplementary Table 2) and impact of immunity upon RDT sensitivity (Supplementary Table 3), whilst holding parameters determining the relationship between EIR and peripheral prevalence at conception and infectious bites during pregnancy fixed to their posterior median (Supplementary Table 1). To focus upon differences associated with choice of strategy we held timing of ANC consistent across all simulations with visits at 20, 27 and 34 weeks gestation for IPTp, ISTp and hybrid strategies, whilst a comparative simulation of Monthly DP was also provided, simulated at 16, 20, 24, 28 and 32 weeks gestation. In addition to uncertainty with respect to the accuracy of the diagnostic we also incorporated uncertainty with respect to the efficacy and longevity of a chosen drug as detailed in Supplementary Table 5.

Supplementary Table 5| Distributions of drug effectiveness used within uncertainty analyses

Parameter	Distribution	Source
Curative efficacy of effective drug (AL, DP or SP in areas with low quintuple mutation)[¥]	Triangle (0.99, 0.98,0.95)[£]	[12]
Half-life of prophylaxis DP or SP in areas with low quintuple mutation.[€]	Triangle(28 days, 21 days,35 days)	[12,13]
Half-life of prophylaxis of AL	Triangle(10 days, 7 days, 15 days)	[9]
Curative efficacy of SP with high quintuple mutation	Triangle(0.79, 0.75, 0.83)	[12]
Half-life of prophylaxis of SP with high quintuple mutation	Triangle (7 days, 0 days, 14 days)	[12]

[¥]Efficacy of DP and AL also assumed to be near perfect so given the same distribution as observed near-perfect curative efficacy of SP in areas of low quintuple

[€]DP and SP assumed to have same prophylactic profile as described in main text

[£]Triangle(m, l, h) refers to a triangle distribution with mode m, lower limit l and upper limit h

References

1. Walker, P. G. T. *et al.* A model of parity-dependent immunity to placental malaria. *Nat. Commun.* **4**, 1609 (2013).

2. Walker, P. G. T., ter Kuile, F. O., Garske, T., Menendez, C. & Ghani, A. C. Estimated risk of placental infection and low birthweight attributable to Plasmodium falciparum malaria in Africa in 2010: a modelling study. *Lancet. Glob. Heal.* **2**, e460-7 (2014).

3. Griffin, J. T. *et al.* Reducing Plasmodium falciparum malaria transmission in Africa: a model-based evaluation of intervention strategies. *PLoS Med.* **7**, 17 (2010).

4. Carnevale, P., Frézil, J. L., Bosseno, M. F., Le Pont, F. & Lancien, J. [The aggressiveness of Anopheles gambiae A in relation to the age and sex of the human subjects]. *Bull. World Health Organ.* **56**, 147–54 (1978).

5. Port, G. R., Boreham, P. F. L. & Bryan, J. H. The relationship of host size to feeding by mosquitoes of the Anopheles gambiae Giles complex (Diptera: Culicidae). *Bull. Entomol. Res.* **70**, 133 (2009).

6. Falk, N. *et al.* Comparison of PCR-RFLP and Genescan-based genotyping for analyzing infection dynamics of Plasmodium falciparum. *Am. J. Trop. Med. Hyg.* **74**, 944–50 (2006).

7. Miller, M. J. Observations on the natural history of malaria in the semi-resistant West African. *Trans. R. Soc. Trop. Med. Hyg.* **52**, 152–68 (1958).

8. Zwang, J. *et al.* Safety and Efficacy of Dihydroartemisinin-Piperaquine in Falciparum Malaria: A Prospective Multi-Centre Individual Patient Data Analysis. *PLoS One* **4**, e6358 (2009).

9. Okell, L. C. *et al.* Contrasting benefits of different artemisinin combination therapies as first-line malaria treatments using model-based cost-effectiveness analysis. *Nat. Commun.* **5**, 5606 (2014).

10. Molineaux, L. & Gramacia, G. The Garki Project: research on the epidemiology and control of malaria in the Sudan savanna of West Africa. (1980).

11. Rogerson, S. J., Mwapasa, V. & Meshnick, S. R. Malaria in pregnancy: linking immunity and pathogenesis to prevention. *Am. J. Trop. Med. Hyg.* **77**, 14–22 (2007).

12. Desai, M. *et al.* Impact of Sulfadoxine-Pyrimethamine Resistance on Effectiveness of Intermittent Preventive Therapy for Malaria in Pregnancy at Clearing Infections and Preventing Low Birth Weight. *Clin. Infect. Dis.* **62**, 323–333 (2016).

13. Okell, L. C. *et al.* The potential contribution of mass treatment to the control of Plasmodium falciparum malaria. *PLoS One* **6**, e20179 (2011).
