# Supplementary material for: Modelling the incremental benefit of introducing malaria screening strategies to antenatal care in Africa
Source: Nat Commun. 2020 Jul 30;11:3799. doi: 10.1038/s41467-020-17528-3 (PMC7393377; doi:10.1038/s41467-020-17528-3)
Supplement: Supplementary file 3 — Reporting Summary [file 41467_2020_17528_MOESM3_ESM.pdf]

nature research

Corresponding author(s): Patrick Walker

Last updated by author(s): Jun 22, 2020

Reporting Summary

Nature Research wishes to improve the reproducibility of the work that we publish. This form provides structure for consistency and transparency in reporting. For further information on Nature Research policies, see Authors & Referees and the Editorial Policy Checklist.

Statistics

For all statistical analyses, confirm that the following items are present in the figure legend, table legend, main text, or Methods section.

n/a	Confirmed	
☐	☒	The exact sample size (n) for each experimental group/condition, given as a discrete number and unit of measurement
☐	☒	A statement on whether measurements were taken from distinct samples or whether the same sample was measured repeatedly
☐	☒	The statistical test(s) used AND whether they are one- or two-sided<br>*Only common tests should be described solely by name; describe more complex techniques in the Methods section.*
☐	☒	A description of all covariates tested
☐	☒	A description of any assumptions or corrections, such as tests of normality and adjustment for multiple comparisons
☐	☒	A full description of the statistical parameters including central tendency (e.g. means) or other basic estimates (e.g. regression coefficient) AND variation (e.g. standard deviation) or associated estimates of uncertainty (e.g. confidence intervals)
☐	☒	For null hypothesis testing, the test statistic (e.g. F, t, r) with confidence intervals, effect sizes, degrees of freedom and P value noted<br>*Give P values as exact values whenever suitable.*
☐	☒	For Bayesian analysis, information on the choice of priors and Markov chain Monte Carlo settings
☒	☐	For hierarchical and complex designs, identification of the appropriate level for tests and full reporting of outcomes
☐	☒	Estimates of effect sizes (e.g. Cohen's d, Pearson's r), indicating how they were calculated

Our web collection on statistics for biologists contains articles on many of the points above.

Software and code

Policy information about availability of computer code

Data collection	No data was collected specifically for this study, analysis was conducted on existing data sets (see Data section)
Data analysis	The majority of data analysis was conducted in R (version 3.4-3.5). The transmission model and associated model of malaria in pregnancy, diagnostic performance and intervention effectiveness was coded and fitted in c++ using Microsoft Visual Studio community versions 2010,2017 and 2019. Source code of the mathematical model developed and used within this analysis, along with a compiled version and compilation and running instructions are available open access at the following repository: www.github.com/patrickgtwalker/malaria_in_pregnancy_istp_model_open

For manuscripts utilizing custom algorithms or software that are central to the research but not yet described in published literature, software must be made available to editors/reviewers. We strongly encourage code deposition in a community repository (e.g. GitHub). See the Nature Research guidelines for submitting code & software for further information.

Data

Policy information about availability of data

All manuscripts must include a data availability statement. This statement should provide the following information, where applicable:

- Accession codes, unique identifiers, or web links for publicly available datasets
- A list of figures that have associated raw data
- A description of any restrictions on data availability

There are three separate primary data sources used in this analysis:
1) Matched RDT and PCR samples from a trial of Intermittent Screening and Treatment in pregnancy based in four countries in West Africa (see reference 8 in the main manuscript for full details)
2) Matched RDT and PCR samples from a trial of Intermittent Screening and Treatment in pregnancy in Western Kenya (see reference 9 in the main manuscript for full details)
3) Matched RDT and PCR samples from a trial of Intermittent Screening and Treatment in pregnancy in Malawi taken from reference (see reference 10 in the main

manuscript for full details)
4) A review of matched RDT and PCR prevalence in non-pregnant adults (see reference 22 in the main manuscript for full details)
Figures 1-3 show data from sources 1-3. Figure S1 and Tables S2-4 show output from fitting to these data. Figure 2c show data from source 4.
Source 1 is available subject to agreement with the original authors from the LSHTM Data Compass https://datacompass.lshtm.ac.uk/4/
Sources 2 and 3 are available for access with the WorldWide Antimalarial Resistance Network (WWARN) at www.WWARN.org. Requests for access will be reviewed by a Data Access Committee to ensure that use of data protects patient privacy according to the terms of consent and ethics approval.
Source 4 is freely available to download from a supplementary data file from https://www.nature.com/articles/nature16039

Field-specific reporting

Please select the one below that is the best fit for your research. If you are not sure, read the appropriate sections before making your selection.

☒ Life sciences ☐ Behavioural & social sciences ☐ Ecological, evolutionary & environmental sciences

For a reference copy of the document with all sections, see nature.com/documents/nr-reporting-summary-flat.pdf

Life sciences study design

All studies must disclose on these points even when the disclosure is negative.

Sample size	Sample sizes were based upon those available from the respective clinical trials.
Data exclusions	No data was excluded unless specified in the main text (e.g. when stratifying by gravidity)
Replication	Full details of model fitting procedure are supplied within the methods and supplementary information. The model itself has also been made available within an open access repository
Randomization	This is not relevant to the study as only the only trial data used were from an intervention arm (intermittent screening and treatment) for model fitting purposes. Measures of goodness of fit are described within the main text.
Blinding	This is not relevant to the study as only the only trial data used were from an intervention arm (intermittent screening and treatment) for model fitting purposes. Measures of goodness of fit are described within the main text.

Reporting for specific materials, systems and methods

We require information from authors about some types of materials, experimental systems and methods used in many studies. Here, indicate whether each material, system or method listed is relevant to your study. If you are not sure if a list item applies to your research, read the appropriate section before selecting a response.

Materials & experimental systems

n/a	Involved in the study	
☒	☐	Antibodies
☒	☐	Eukaryotic cell lines
☒	☐	Palaeontology
☒	☐	Animals and other organisms
☒	☐	Human research participants
☒	☐	Clinical data

Methods

n/a	Involved in the study	
☒	☐	ChIP-seq
☒	☐	Flow cytometry
☒	☐	MRI-based neuroimaging
